# Genetic Interaction between Arabidopsis *Qpm3.1* Locus and Bacterial Effector Gene *hopW1-1* Underlies Natural Variation in Quantitative Disease Resistance to *Pseudomonas* Infection

**DOI:** 10.3389/fpls.2017.00695

**Published:** 2017-05-04

**Authors:** Qi Luo, Wei-Wei Liu, Ke-Di Pan, You-Liang Peng, Jun Fan

**Affiliations:** ^1^Ministry of Agriculture Key Laboratory of Plant Pathology, College of Plant Protection, China Agricultural UniversityBeijing, China; ^2^State Key Laboratory of Agrobiotechnology, China Agricultural UniversityBeijing, China

**Keywords:** Arabidopsis, *hopW1-1*, natural variation, *Pseudomonas syringae*, quantitative disease resistance, quantitative trait locus

## Abstract

Wide quantitative variation in plant disease resistance across Arabidopsis wild populations has been documented and the underlying mechanisms remain largely unknown. To investigate the genetic and molecular basis of this variation, Arabidopsis recombinant inbred lines (RILs) derived from Aa-0 × Col-0 and Gie-0 × Col-0 crosses were constructed and used for inoculation with *Pseudomonas syringae* pathovars *maculicola* ES4326 (ES4326) and *tomato* DC3000 (DC3000). Bacterial growth assays revealed continuous distribution across the large differences between the most and the least susceptible lines in the RILs. Quantitative trait locus (QTL) mapping analyses identified a number of QTLs underpinning the variance in disease resistance, among which *Qpm3.1*, a major QTL on chromosome III from both Aa-0 and Gie-0 accessions, preferentially restricted the growth of ES4326. A genetic screen for the ES4326 gene selectively leading to bacterial growth inhibition on accession Aa-0 uncovered the effector gene *hopW1-1*. Further QTL analysis of disease in RILs inoculated with DC3000 carrying *hopW1-1* showed that the genetic interaction between *Qpm3.1* and *hopW1-1* determined Arabidopsis resistance to bacterial infection. These findings illustrate the complexity of Arabidopsis-*Pseudomonas* interaction and highlight the importance of pathogen effectors in delineating genetic architectures of quantitative variation in plant disease resistance.

## Introduction

Plants rely on a complex innate immune system to ward off pathogen attacks. Perception of pathogen-associated molecular patterns (PAMPs) by plant cell surface distributed receptors elicits the first line of defense-PAMP triggered immunity (PTI). To succeed in infection pathogens secrete effectors to the apoplast or inside plant cells to suppress PTI and reprogram host physiology in favor of disease, a process often referred to as effector-triggered susceptibility (ETS) (Jones and Dangl, 2006; Toruno et al., 2016). Plants have evolved a second line of defense, also known as effector-triggered immunity (ETI), by encoding diverse resistance (R) proteins to detect such effectors and activating high levels of immune responses such as hypersensitive cell death (Jones and Dangl, 2006). R gene-mediated resistance is typically qualitative and effective to restrict pathogen strains carrying specific effectors (avirulence proteins, Avr); in absence of a successful R-Avr interaction, however, quantitative disease resistance reduces the growth and spread of virulent pathogens, thereby protecting plants from further infection (Dangl and Jones, 2001; Poland et al., 2009).

The phenomena of quantitative disease resistance, also referred to as partial disease resistance or basal resistance, has been widely observed in many plant pathosystems, in which the distribution of resistance values in a genetically segregating population is typically continuous and does not follow a Mendelian pattern of inheritance (Poland et al., 2009). Genetic dissection of quantitative disease resistance in many crop plants with quantitative trait locus (QTL) mapping has uncovered genetic loci conferring resistance to a wide range of diseases (St. Clair, 2010). Recently, a number of genes mediating quantitative disease resistance have been successfully cloned from major crop plants including wheat (Fu et al., 2009; Krattinger et al., 2009), rice (Fukuoka et al., 2009; Hayashi et al., 2010), maize (Hurni et al., 2015; Zuo et al., 2015) and soybean (Cook et al., 2012). Notably, many of these genes provide durable resistance in the field and encode proteins distinct to typical R proteins, indicating that quantitative disease resistance and R-gene mediated resistance may recruit different components to deploy.

The model plant Arabidopsis has been used intensively to investigate the genetic and molecular basis of disease resistance to virulent pathogens with diverse lifestyles. Major progress has been achieved on how the signal molecules including salicylic acid (SA), jasmonic acid, and ethylene orchestrate plant defenses (Pieterse et al., 2012). In addition, multiple pattern recognition receptors have been uncovered in the plant to elicit PTI in response to various PAMPs (Gómez-Gómez and Boller, 2000; Zipfel et al., 2006; Miya et al., 2007; Albert et al., 2015). Nevertheless, most of these findings are based on experiments with frequently used Arabidopsis accessions such as Col-0. Several studies have demonstrated wide quantitative variation in disease resistance across Arabidopsis accessions collected from wild populations at different geographical locations, and many accessions are much less susceptible than Col-0 to pathogen infection, indicating the presence of important unknown mechanisms mediating disease resistance in these accessions (Kover and Schaal, 2002; Fan et al., 2008). Therefore, further investigation on the basis of this variation will not only broaden our knowledge on the complex genetic architectures of plant defense networks but also may help us design crops with durable resistance to a broad range of phytopathogens.

The bacterial effector HopW1-1, also known as HopPmaA, has been found in several strains of *Pseudomonas syringae* pathovars (pv) *maculicola* and *phaseolicola*, but is absent from the strain *P. syringae* pv *tomato* DC3000 (DC3000) (Guttman et al., 2002; Lindeberg et al., 2006). When delivered into plant cells by *P*. *syringae*, HopW1-1 targets the actin cytoskeleton and inhibits endocytosis, thereby promoting bacterial virulence in susceptible Arabidopsis plants (Kang et al., 2014). However, in some accessions such as Ws, *hopW1-1* restricts bacterial growth and elicits resistance responses including the accumulation of SA (Lee et al., 2008). Three HopW1-1-interacting (WIN) proteins have been identified from Arabidopsis. Overexpression or downregulation of WIN proteins has diverse effects on plant disease resistance induced by HopW1-1 or other bacterial effectors, indicating that WIN proteins may have different roles in modulating bacteria-plant interactions (Lee et al., 2008). Variation in resistance among Arabidopsis accessions to bacterial strains carrying *hopW1-1* has been revealed in several studies (Lee et al., 2008; Rant et al., 2013), but the genetic region underlying plant resistance specific to HopW1-1 remains to be revealed.

In this study, Arabidopsis accessions Aa-0 and Gie-0 were found to support different levels of bacterial growth compared with Col-0 plants. Two populations of recombinant inbred lines (RILs) derived from Aa-0 × Col-0 and Gie-0 × Col-0 crosses were constructed and used for genetic dissection of the variance in disease resistance to bacterial strain *P. syringae* pv *maculicola* ES4326 (ES4326) and DC3000, respectively. QTL analyses of the levels of bacterial growth continuously distributed across these two RILs showed that genetic variation in Aa-0 and Gie-0 at a locus on chromosome III, designated as *Qpm3.1*, preferentially restricted the growth of bacterial strain ES4326. Further screen for the genetic factor in ES4326 that limited bacterial growth on these Arabidopsis accessions successfully identified the effector gene *hopW1-1*. These findings highlight the role of pathogen effectors in quantitative natural variation in plant resistance to bacterial infection. The possible mechanism is also discussed on how *Qpm3.1*-*hopW1-1* interaction led to enhanced resistance.

## Materials and Methods

### Plant Materials and Growth Conditions

Arabidopsis accessions Aa-0, Gie-0, and Col-0 were used in this study. To construct RILs, F2 plants derived from Aa-0 × Col-0 and Gie-0 × Col-0 crosses with Col-0 as the female parent were self-fertilized and taken to F9 generation by single-seed descent method. Seeds of F9 plants were collected for subsequent disease assays. Heterogeneous inbred families (HIFs) were obtained by self-crossing the F6-RIL lines heterogeneous at markers cosegregating with *Qpm3.1* locus, 1.3Mb and NGA172, in Col-Aa and Col-Gie populations, respectively. Arabidopsis seeds were incubated in 0.1% of agarose at 4°C for 2 days to synchronize germination. Seedlings were grown in a growth room under a 9-h photoperiod at 23°C.

### Bacterial Strains and Disease Assay

The wild type and *luxCDABE*–tagged *P. syringae* strains ES4326-lux and DC3000-lux were grown on King’s B plates containing kanamycin (25 μg ml^-1^) and rifampicin (25 μg ml^-1^) as described (Fan et al., 2008). To inoculate Arabidopsis, bacterial suspensions prepared from overnight grown plates were diluted to levels as indicated and pressure-infiltrated with a needleless syringe into freshly expanded leaves of five-week-old Arabidopsis plants. To get a larger difference in bacterial growth between Col-0 and Aa-0, plants were covered after the inoculation, and for the same reason, plants were not covered when Col-0 and Gie-0 accessions were analyzed for variation in bacterial growth. To perform phenotypic analyses efficiently, bioluminescence assay was used to determine the bacterial growth on RILs (Fan et al., 2008). Briefly, leaf discs of 5 mm in diameter were excised from the inoculated leaves and put into 96-well plates to measure luminescence with the MP180 detector (Beijing Yikang Baifang Co., Ltd.). For each line, at least three individual plants were inoculated and four disks of each plant were measured. Colony counting method was also used to analyze bacterial number in leaf disks (Whalen et al., 1991).

### Construction of Linkage Maps and QTL Mapping

Genomic DNA was extracted from F9 seedlings of each RIL, and the resulting DNA samples were amplified with SSLP and dCAPS (Supplementary Tables S2, S3) primer pairs in 10 μl PCR reactions. PCR products (for SSLP markers) or enzyme-digested PCR products (for dCAPS markers) were resolved by agarose gel electrophoresis (3%, w/v). Genotype data of RILs were analyzed by QTL IciMapping software and the algorithm of nnTwoOpt was chosen to locate markers on the chromosomes (Meng et al., 2015). Levels of bioluminescence of inoculated leaf disks of a RIL plant were subjected to logarithmic transformation and then an average of the resulting data was taken as the value for the disease phenotype of the line. Phenotypes of all the RILs were analyzed with the same software and the method of ICIM-Add was used to calculate the additive effect, while the method of ICIM-EPI was used to check the possible interactions among the QTLs. The threshold for statistical significance (95% confidence interval) of LOD was calculated by permutation test (*n* = 1000). The amount of heritability (Hˆ2) was calculated by ANOVA in QTL IciMapping software.

### Construction of Cosmid Genomic Library of *P. syringae* pv. *maculicola* ES4326

The cosmid vector pLAFR5 (Keen et al., 1988) was digested with *Bam*HI followed by dephosphorylation and *Sca*I digestion. Meanwhile, size-fractionated (18-27 Kb) genomic DNA fragments of ES4326 were generated via partial digestion with *Sau*3AI and retrieved from 0.7% agarose gel as previously described (Zhen and Swank, 1993). The DNA fragments were then ligated into the *Bam*HI site of pLAFR5 and packaged into lambda phage using the MaxPlax^TM^ Lambda Packaging Extracts (Epicentre, Wisconsin, USA) according to manufacturer’s protocol. After infection of *E. coli* DH5α strain with the packaged products, bacterial cells were plated on King’s B agar medium supplemented with tetracycline (10 μg ml^-1^) and incubated overnight at 37°C. Colonies were picked and stored at –70°C to build up the library. The cosmid clones in DH5α were subsequently mobilized into the DC3000-lux strain by triparental mating with the help of plasmid pRK2013 using 96-well plates. The exconjugants were recovered on King’s B agar plates supplemented with tetracycline (5 μg ml^-1^), kanamycin (15 μg ml^-1^), and rifampicin (25 μg ml^-1^) after incubation for 3–5 days at 28°C.

### Library Screening for Cosmids that Reduce Virulence of DC3000-Lux Specifically on Aa-0 and Cloning of the *hopW1-1*

The DC3000-lux clones bearing the ES4326 genomic cosmid library were used to inoculate plants of Arabidopsis accessions Aa-0 and Col-0. By using luminescence assay, clones growing poorly on Aa-0 but normally on Col-0 plants, compared with the growth of DC3000-lux, were selected and saved for further analysis. Cosmids of the selected clones were extracted and transformed into *E. coli* for propagation. The resulting cosmids were isolated and sequenced with M13 primers to obtain the sequences of insert ends.

The *hopW1-1* gene with native promoter was cloned through Gateway recombinational technology. A pair of primers 5′-AAAAAGCAGGCTCAAAGGTTGGAGTCTCCGAGAAAC-3′ and 5′- AGAAAGCTGGGTCGATGCAGACCTGGCGTATCATAT-3′ were used to amplify the genomic DNA of ES4326. The PCR product was subsequently amplified with primers that reconstitute the complete attB sites and cloned into a modified pME6031 vector compatible with Gateway cloning, which can be stably maintained in DC3000 (Heeb et al., 2000).

## Results

### Natural Variation in Disease Resistance of Arabidopsis Accessions Against Diverse *P. syringae* Pathovars

**FIGURE 1:**
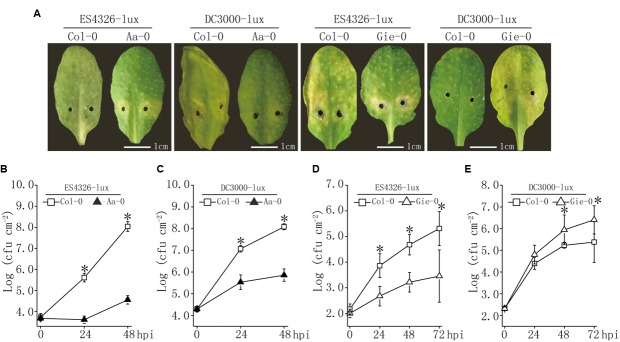
**Variation in disease resistance to *Pseudomonas syringae* among Arabidopsis accessions Aa-0, Gie-0, and Col-0. (A)** Disease symptoms on leaves infected with lux-tagged *P*. *syringae* pathovars *maculicola* ES4326 (ES4326-lux) or *tomato* DC3000 (DC3000-lux). **(B–E)** Bacterial growth *in planta* after inoculation with ES4326-lux or DC3000-lux. Bacterial suspensions were pressure infiltrated into fully expanded leaves of 4∼5 week old Arabidopsis plants using a 1 ml needleless syringe. Bacterial inocula of optical density (OD_600_) at 0.003 and 0.0002 were used to compare the difference in disease levels between Col-0 and Aa-0, and between Col-0 and Gie-0 plants, respectively. After inoculation, Col-0/Aa-0 plants were covered to keep high relative humidity, whereas Col-0/Gie-0 plants were left uncovered. Pictures of inoculated leaves were taken 48 and 72 hours post inoculation (hpi) for Col-0/Aa-0 and Col-0/Gie-0 plants, respectively. Asterisks indicate significant differences between the genotypes (Student’s *t*-test, *P* < 0.05). Data shown are means ± SD (*n* = 8). Experiments were repeated three times with similar results.

Our and others’ studies have documented substantial variation in disease resistance to *P. syringae* infection among Arabidopsis natural populations (Kover and Schaal, 2002; Perchepied et al., 2006; Fan et al., 2008; Hossain and Sultana, 2015). Based on our previous work (Fan et al., 2008), we used two Arabidopsis accessions, Aa-0 and Gie-0, in combination with the reference accession Col-0, to further investigate the genetic and molecular basis of this variation. We first characterized the disease outcome of the interactions between these accessions and two bacterial strains. Results showed that 48 hours after inoculation of the plants with *P. syringae* strains DC3000-lux and ES4326-lux, leaf tissues of Col-0 plants developed clear disease symptoms for both strains, whereas Aa-0 plants displayed only weak chlorosis closely around injection sites for ES4326-lux and hardly any visible symptom for DC3000-lux (**Figure 1A**). Consistent with the symptom observed, bacterial counts of ES4326-lux and DC3000-lux in Aa-0 leaf tissue distant to the injection sites were about three and two orders of magnitude lower than that in Col-0 leaves, respectively (**Figures 1B,C**), indicating that Aa-0 plants were significantly more resistant to both strains than Col-0 plants.

Similar to accession Aa-0, Gie-0 plants displayed attenuated symptom to ES4326-lux infection and supported two orders of magnitude lower bacterial growth than Col-0; however, for DC3000-lux inoculation, the disease symptom was enhanced and bacterial counts were 10-fold higher in Gie-0 than that in Col-0 plants (**Figures 1A,D,E**). These observations demonstrated that the natural variation in disease resistance was not only associated with Arabidopsis accessions but also influenced by the invading bacterial strains.

### Natural Variation in Disease Resistance of Arabidopsis to Bacterial Infection was Quantitatively Determined

**FIGURE 2:**
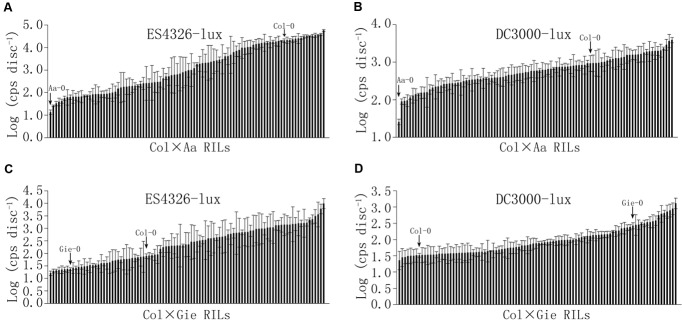
**Continuous distribution of sizes of bacterial population among Col × Aa and Col × Gie RILs.** Bacterial inocula of optical density (OD_600_) at 0.003 and 0.0002 were used for Col × Aa RILs and Col × Gie RILs, respectively. Leaf discs from four leaves per line were taken to measure luminescence (photon counts per second) at 48 hpi for Col × Aa RILs **(A,B)** and 72 hpi for Col × Gie RILs **(C,D)**. Bacterial titers of parents are indicated by arrows. The experiment was repeated three times and the average levels of luminescence of each line from three experiments are shown. Error bars denote standard deviations (*n* = 3).

To further investigate genetics underlying the variation in disease resistance between these Arabidopsis accessions, we constructed two populations of RILs derived from Col-0 × Aa-0 and Col-0 × Gie-0 crosses. Ninety-six individual lines of each F9 population were inoculated with ES4326-lux or DC3000-lux, and bioluminescence assay of bacterial growth revealed continuous distribution between the most and the least susceptible lines in the two RIL populations (**Figure 2**, Supplementary Figure S1 and Table S1), which strongly indicated that variation in disease resistance was quantitative and likely the result of multigene-effect. Transgressive segregation could also be observed among the two tested RIL populations (**Figure 2**), suggesting that parents of each RIL population may carry genes associated with quantitative disease resistance at different genetic loci.

### QTL Mapping Revealed Genetic Loci with Large Effect on Arabidopsis Resistance to Bacterial Infection

**FIGURE 3:**
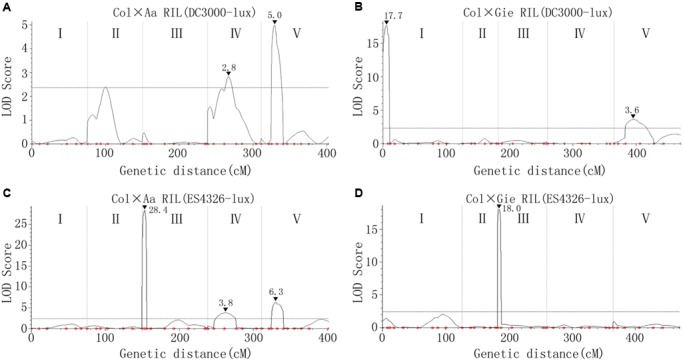
**Arabidopsis quantitative trait loci (QTLs) controlling variation in disease resistance to *P. syringae* infection. (A,B)** QTLs underlying the resistance variance to DC3000-lux in Col × Aa **(A)** and Col × Gie **(B)** RILs. **(C,D)** QTLs underlying the resistance variance to ES4326-lux in Col × Aa **(C)** and Col × Gie **(D)** RILs. QTL analyses are based on genotypic data and levels of bacterial luminescence of the RILs. The curved lines indicate likelihood statistics (scores of the likelihood of odds, LOD) for the positions of QTLs and the significance threshold at 95% confidence interval calculated by permutation tests (*n* = 1000) is plotted as horizontal lines. I–V denote five individual linkage groups. Positions of the markers used for analysis are indicated as small dots. Peaks of LOD scores are indicated by filled triangles along with the values.

Simple sequence length polymorphism (SSLP) markers and derived cleaved amplified polymorphic sequence (dCAPS) markers (Supplementary Tables S2, S3) were used to analyze the genotype of each line of the two RIL populations to construct linkage maps used for QTL analysis (Supplementary Figure S2). The QTL analysis uncovered several Arabidopsis genomic regions dominating the variation in disease resistance to ES4326-lux (QTL to *P. syringae* pv. *maculicola*, *Qpm*) and DC3000-lux (QTL to *P. syringae* pv. *tomato*, *Qpt*). The amount of heritability (Hˆ2) for DC3000-lux growth in Col × Aa and Col × Gie RILs was 0.76 and 0.88, respectively, whereas that for ES4326-lux growth in Col × Aa and Col × Gie RILs was 0.88 and 0.69, respectively. For DC3000-lux, a QTL on chromosome V that conferred nearly a quarter of the variance in resistance was identified in Col × Aa RILs, and another QTL at the top arm of chromosome I was revealed in Col × Gie RILs, accounting for 60% of the variance in resistance (**Figures 3A,B** and **Table 1**). Analysis of ES4326-lux infected RILs, however, revealed a region on chromosome III highly overlapped between both RILs, accounting for over 60% of the variance in resistance (**Figures 3C,D** and **Table 1**). In addition, a number of QTLs with smaller effect were also detected in the tested RIL and bacterial interactions (**Table 1**).

**Table 1:** Summary of Arabidopsis quantitative trait locus (QTL) conferring resistance to lux tagged *Pseudomonas syringae* pv. *tomato* DC3000 (*Qpt*) and pv. *maculicola* ES4326 (*Qpm*).

QTL name and chromosome	Position (cM)	Nearest genetic marker	Confidence interval (cM)	LOD^a^	Additive effect	*R*^2^ (%)^b^
*Qpt1.1*	6.0	3611696	0–8.0	17.7	+0.3	60.0
*Qpt4.1*	28.0	6907866	22.6–38.6	2.8	+0.1	16.0
*Qpt5.1*	18.0	NGA139	13.6–20.9	5.0	+0.2	25.4
*Qpt5.2*	30.0	NGA76	18.0–40.9	3.6	+0.1	12.1
*Qpm3.1*_Aa_	3.0	1.3Mb	0–5.5	28.4	+0.8	64.8
*Qpm3.1*_Gie_	2.0	NGA172	0–5.3	18.0	+0.5	62.1
*Qpm4.1*	25.0	6907866	22.6–38.6	3.8	+0.2	4.9
*Qpm5.1*	19.0	NGA139	13.6–20.9	6.3	+0.3	8.9

*^a^Significant QTLs with LOD score ≥ threshold based on permutation test.*

*^b^Percentage of phenotypic variation explained by QTL.*

**FIGURE 4:**
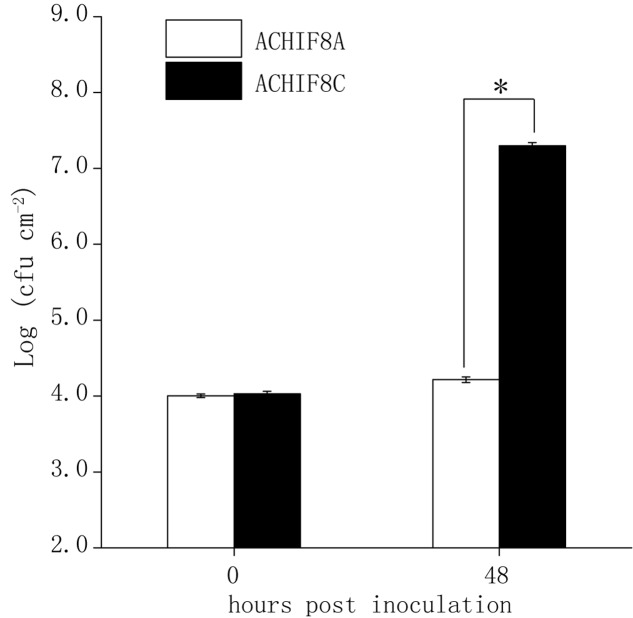
**Validation of the major QTL *Qpm3.1* in Aa-0 with the heterogeneous inbred family (HIF).** Forty-eight individuals of a Col × Aa HIF (ACHIF8) were inoculated with ES4326-lux at OD_600_ = 0.003. The inoculated plants were covered to keep high relative humidity. Genotypes of individual plants were determined with the tightly associated marker 1.3 Mb and sizes of the bacterial population were compared between plants with homozygous Aa-0 (ACHIF8A) and plants with homozygous Col-0 (ACHIF8C) genotypes. Data shown are means ± SD (*n* = 8). The asterisk indicates a significant difference between treatments (Student’s *t*-test, *P* < 0.05). The experiment was repeated three times with similar results.

To test if the detected major QTLs on chromosome III (*Qpm3.1_Aa_* and *Qpm3.1_Gie_*) were truly conferring the variation in resistance to ES4326-lux, heterogeneous inbred families (HIFs) were obtained by selfing F6-RIL plants heterozygous at the QTL, and the resultant HIFs, ACHIF8 (from Col × Aa RILs) and GCHIF10 (from Col × Gie RILs), were subsequently analyzed for resistance to ES4326-lux. Results showed that plants with homozygous Aa-0 genotype at marker 1.3Mb or with Gie-0 genotype at marker NGA172 were significantly more resistant than those with homozygous Col-0 genotype (**Figure 4** and Supplementary Figure S3), indicating that the variation in disease resistance was tightly linked to the genetic variation at this locus. We thus designate this highly overlapped region as *Qpm3.1*.

### Identification of the Effector Gene *hopW1-1* from Pathovar Maculicola that Reduced Bacterial Growth in Arabidopsis Accession Aa-0 and Gie-0

**FIGURE 5:**
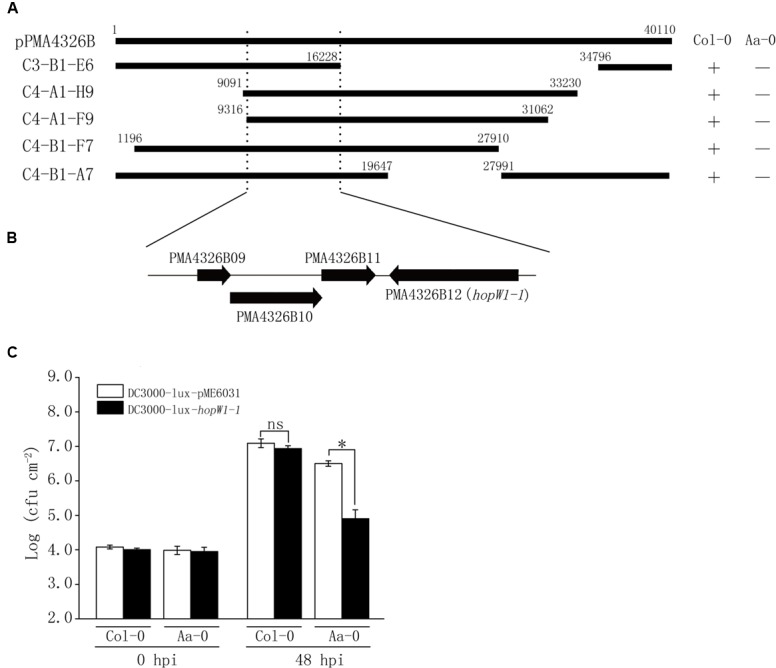
**Identification from ES4326 of the effector gene *hopW1-1* that selectively reduced the bacterial growth on Aa-0. (A)** Cosmid clones capable of reducing bacterial growth on Aa-0 share a common region of pPMA4326B from ES4326. Top bar denotes the linearized pPMA4326B in the full length of 40,110 base pairs. Each cosmid is marked with numbers showing the positions of insert ends. “+” indicates that the growth of bacterial cosmid clone *in planta* was similar to that of empty vector control. “–” indicates that the growth of bacterial cosmid clone *in planta* was significantly reduced compared with vector control. **(B)** Annotated genes in the minimal region of pPMA4326B shared by all five cosmid clones. **(C)** The effector gene *hopW1-1* selectively reduced the growth of DC3000-lux in Aa-0 but not Col-0 plants. Plants were pressure inoculated with bacteria at OD_600_ = 0.01. Bacterial growth was determined by counting colony forming unit (cfu) of three leaf disks from each plant and at least four plants were used in each treatment. Data shown are means ± SD (*n* = 12). The asterisk indicates a significant difference between treatments (Student’s *t*-test, *P* < 0.05). “ns” denotes no significant difference. This experiment was repeated three times with similar results.

The above results showed that levels of ES4326-lux growth on Aa-0 and Gie-0 accessions were significantly lower compared to DC3000-lux (**Figure 1**) and that the prominent QTL *Qpm3.1* appeared to confer resistance specifically to strain ES4326-lux and was ineffective when DC3000-lux was used for inoculation (**Figure 3**). Therefore, we hypothesized that certain genetic component in bacteria may also be involved in the quantitative disease resistance of Arabidopsis plants. To search for this putative genetic factor we constructed a cosmid library of ES4326 genomic DNA and mobilized the resultant library into DC3000-lux by conjugation to screen for clones impaired in virulence on Aa-0 but not on Col-0 plants. Out of nearly 500 clones screened, five cosmids were found to be capable of reducing the growth of DC3000-lux preferentially on Aa-0 plants. The resulting cosmids share a 7 kb region of the plasmid B of ES4326 (**Figure 5A**), in which an effector gene *hopW1-1* has been described previously (**Figure 5B**) (Guttman et al., 2002; Lee et al., 2008). Subsequently, we cloned the *hopW1-1* and found that plasmids harboring this gene significantly attenuated growth of DC3000-lux on Aa-0 and Gie-0, but not on Col-0 plants (**Figure 5C** and Supplementary Figure S4), suggesting that *hopW1-1* may be a key player sufficient in conditioning disease resistance in Arabidopsis to ES4326.

### Genetic Interaction between *Qpm3.1* and *hopW1-1* Underpinned Arabidopsis Quantitative Disease Resistance to Bacterial Infection

**FIGURE 6:**
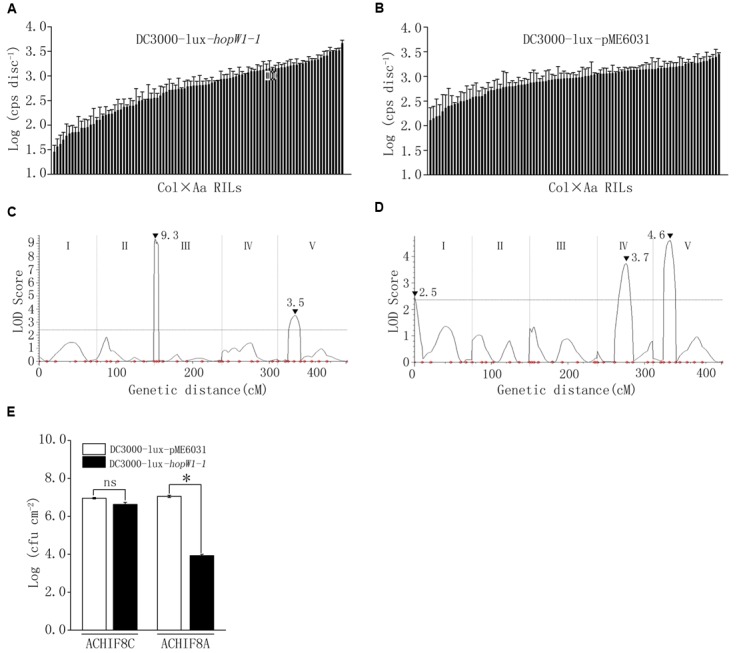
**The interaction between *Qpm3.1* and *hopW1-1* contributed to the quantitative disease resistance. (A,B)** Continuous distribution of luminescence levels of DC3000-lux-*hopW1-1* **(A)** and DC3000-lux-pME6031 **(B)** in Col × Aa RILs. Leaves were pressure inoculated with the bacteria at OD_600_ = 0.003. Inoculated plants were covered and four leaf discs from each plant were sampled 48hpi for luminescence assay. Data shown are means of results of three repeated experiments. Error bars denote standard deviations (*n* = 3). **(C,D)** QTLs mediating quantitative resistance to DC3000-lux-*hopW1-1* **(C)** and DC3000-lux-pME6031 **(D)** in Col × Aa RILs. Peak LOD scores are indicated by filled triangles along with their values. **(E)** Genetic interaction between *Qpm3.1* and *hopW1-1* restricted bacterial growth *in planta*. Individuals of HIF family ACHIF8 were genotyped with marker 1.3 Mb tightly associated with locus *Qpm3.1*. Plants with Aa-0 genotype (ACHIF8A) and plants with Col-0 genotype (ACHIF8C) were pressure infiltrated with DC3000-lux-*hopW1-1* and DC3000-lux-pME6031 at OD_600_ = 0.0005. The inoculated plants were covered to keep high relative humidity. Levels of the bacterial population were determined at 72 hpi. Data shown are means ± SD (*n* = 8). The asterisk indicates a significant difference between treatments (Student’s *t*-test, *P* < 0.05). “ns” denotes no significant difference. This experiment was repeated three times with similar results.

To further elucidate the genetic component in Arabidopsis involved in the interaction with *hopW1-1*, DC3000-lux transformed with *hopW1-1* (DC3000-lux-*hopW1-1*) or empty vector (DC3000-lux-pME6031) were used to inoculate Col-Aa and Col-Gie RILs. Luminescence assay revealed continuous distribution of bacterial growth within each RIL population (**Figures 6A,B**, Supplementary Figure S5A, and Table S1), and QTL analysis uncovered prominent extra peaks at *Qpm3.1* locus specifically associated with infections by strain DC3000-lux-*hopW1-1* (compare **Figure 6C** with **Figure 6D**, and **Figure 3B** with Supplementary Figure S5B), indicating that the genetic interaction between *Qpm3.1* and *hopW1-1* may determine the resistance in Arabidopsis against bacterial infection. This speculation was further corroborated by observations that plants carrying *Qpm3.1* in ACHIF8 family were significantly more resistant to strain DC3000-lux*-hopW1-1*, but not to DC3000-lux strain with the empty vector (**Figure 6E**). However, the *Qpm3.1* mediated resistance was substantially compromised when higher bacterial inocula were used for infection (compare **Figure 6E** with Supplementary Figure S6).

### The Epistatic Effect of Loci *Qpm3.1* and *Qpt1.1* on Variation in Quantitative Disease Resistance was hopW1-1 Dependent

**FIGURE 7:**
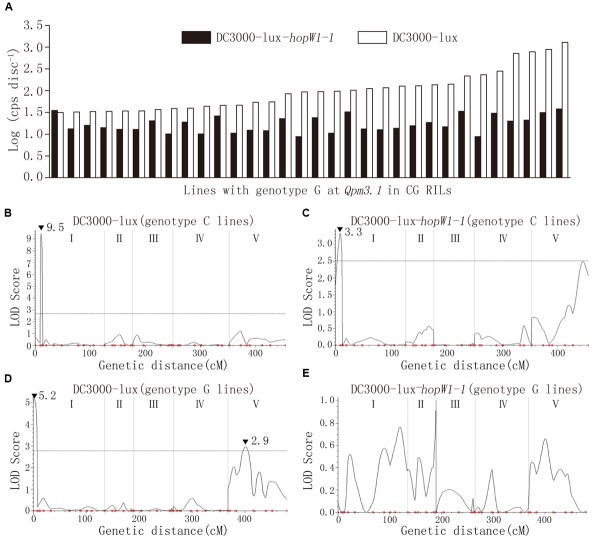
**The epistatic effect between *Qpm3.1* and *Qpt1.1*. (A)** Comparison of the variation in levels of the bacterial population between DC3000-lux strains with or without the effector gene *hopW1-1* on Col × Gie RILs carrying *Qpm3.1*. **(B–E)** QTL analysis of resistance variation in subgroups of Col × Gie RILs. Col × Gie RILs were inoculated with DC3000-lux strains with or without *hopW1-1*. Plant lines with genotypes homozygous for Col-0 **(B,C)** or Gie-0 **(D,E)** at markers NGA172 and NGA76 juxtaposing *Qpm3.1* were subgrouped and used for QTL analysis. Peak LOD scores are indicated by filled triangles along with their values.

In Col × Gie RILs major QTLs *Qpm3.1* and *Qpt1.1* were detected to condition variation in resistance to ES4326 and DC3000, respectively (**Figures 3B,D**). Interestingly, however, the peak of *Qpt1.1* was absent and only *Qpm3.1* was detected when DC3000-lux-*hopW1-1* was used to inoculate the RILs (Supplementary Figure S5B), which strongly indicated the existence of epistasis between the two QTLs. We compared levels of bacterial growth *in planta* and found that on RILs with the *Qpm3.1* genotype the variation in population size was apparently reduced when *hopW1-1* was present in DC3000-lux (**Figure 7A**). Subsequently, Col × Gie RILs were subgrouped into lines with or without *Qpm3.1* and used for QTL analysis of its impact on *Qpt1.1*. As shown in **Figure 7**, on lines without *Qpm3.1* (Col-0 genotype at the locus) the peak of *Qpt1.1* was detectable regardless of the presence of *hopW1-1* in DC3000-lux (**Figures 7B,C**); however, on lines with *Qpm3.1* the peak of *Qpt1.1* was only detectable when *hopW1-1* was absent in bacteria (**Figure 7D**), and the effector gene completely abolished the *Qpt1.1-*mediated variation in bacterial levels of DC3000-lux (**Figure 7E**). Thus, the effect of *Qpm3.1* is epistatic to *Qpt1.1* and the epistasis requires bacterial *hopW1-1*.

## Discussion

In this study, we identified several QTLs mediating quantitative disease resistance to the lux-tagged DC3000 and ES4326 strains, respectively (**Figure 3** and **Table 1**). The patterns of QTL distribution apparently differed in association with the strains used for inoculation. Among *Qpm* loci, the prominent effect of *Qpm3.1* was detected from both Col × Aa and Col × Gie RILs challenged with ES4326 but was absent when inoculated with DC3000. This locus also coincides with the major QTL *QRps.JIC-3.1* that has been identified during infection of Col × Ler RILs with the strain ES4326 (Rant et al., 2013), indicating that these two QTLs may be the same and potentially strain-specific and broadly exist in Arabidopsis accessions.

In contrast, the distribution of *Qpt* loci seemed largely distinct between the two RILs although marginal overlap of genetic region exists between *Qpt5.1* and *Qpt5.2* (**Figure 3** and **Table 1**), suggesting that the genetic basis dominating variance in resistance of Aa-0 and Gie-0 against virulent DC3000 differed substantially. The strain DC3000 has been used in other studies for dissection of natural variation in disease resistance in Arabidopsis, and multiple major QTLs have been uncovered to restrict the size of the bacterial population *in planta*. The genetic location of *Qpt5.1*, one of the large effect QTLs detected in this study, is close to that of *PRP-Ps2*, a major QTL identified with the population of Bayreuth × Shahdara RILs (Perchepied et al., 2006), but is distant from another major QTL previously identified at the bottom of chromosome V that explains most of the variation in resistance between accessions Columbia and San Feliu-2 (Kover and Cheverud, 2007). The location of *Qpt1.1*, another major QTL identified in this study determining the variation in susceptibility between Col-0 and Gie-0, is clearly distinct from the QTL previously identified on the same chromosome (Fan et al., 2008). These studies demonstrate the complexity of natural variation in Arabidopsis resistance to strain DC3000. We expect that more major QTLs could be uncovered when other accessions or alternative inoculation methods are used for analysis.

Notably, we also found that genetic regions closely overlap between *Qpt4.1* and *Qpm4.1*, and between *Qpt5.1* and *Qpm5.1* as well (**Table 1**), implying the existence of QTLs effective against both bacterial strains. However, it remains to be elucidated whether the same genes in the overlapped QTLs mediate disease resistance to both strains. The diversity of QTLs detected in disease resistance, the sensitivity of *Qpm3.1* to higher bacterial inocula (Supplementary Figure S6), and the epistasis between *Qpm3.1* and *Qpt1.1* (**Figure 7**) revealed in this study collectively indicate that the genetic architecture of the quantitative disease resistance is highly complex and further molecular dissection is necessary to unravel the underlying mechanisms.

In Arabidopsis, the molecular basis of the quantitative natural variation in disease resistance to bacterial infection is still poorly understood since few of the identified QTLs has been cloned. It has been proposed that PTI, ETI, and ETS collectively determine the plant resistance to a virulent pathogen (Jones and Dangl, 2006). Hence, the genetic variation modulating the outcome of any of these processes may lead to the quantitative variation in disease resistance. It has been shown that natural allelic variation at the locus *ACCELERATED CELL DEATH 6* causes marked differences in accumulation of SA and in resistance to infections by a panel of phylogenetically distinct pathogens including *P. syringae* pv *tomato* DC3000 (Todesco et al., 2009). Other studies have shown that disruption of genes required for ETI compromises resistance in Arabidopsis against both virulent and non-adapted of *P. syringae* strains, indicating that ETI protects the plant during these seemingly distinct interactions (Zhang et al., 2010). In addition, the genetic variation at locus *FLS2*, which encodes the flagellin receptor that mediates PTI responses, has also been shown to account for the variation in Arabidopsis resistance to non-adapted *P. syringae* pv *phaseolicola* (Forsyth et al., 2010). A more recent study discovered that natural variation in Arabidopsis responses to the type III effector HopAM1 secreted by disarmed DC3000 is quantitatively determined although the HopAM1-induced cell death and chlorosis do not affect bacterial growth *in planta* (Iakovidis et al., 2016). Our study clearly demonstrated that the locus *Qpm3.1* was effective to restrict the growth of bacterial strains carrying the effector gene *hopW1-1*, a reminiscent of specificity commonly exhibited in R-gene mediated resistance. Several genes around the *Qpm3.1* locus are indeed annotated to encode R-like proteins, including one TIR-NBS-LRR gene (*At3g04220*), one TIR-NB gene (*At3g04210*); and four receptor-like kinase/protein genes (*At3g05360*, *At3g05370*, *At3g05650*, and *At3g05660*); several major R-genes have been shown to be responsible for race-specific QTLs conditioning quantitative resistance to *Xanthomonas* bacteriain Arabidopsis and rice plants (Li et al., 1999; Debieu et al., 2015). However, the narrow spectrum of *Qpm3.1* does not necessarily mean that a “defeated” or “weak” R-gene is responsible for the observed quantitative resistance (see discussion below) as diverse molecular mechanisms other than R*-avr* interaction have been found to underpin the quantitative resistance in multiple pathosystems (Poland et al., 2009; French et al., 2016). In fact, apart from the R-like genes mentioned above, 12 more genes are annotated to confer defense responses among the predicted 384 genes within the *Qpm3.1* locus. It is interesting to observe that higher inocula (OD_600_ 0.03) compromised *Qpm3.1s* mediated resistance (Supplementary Figure S6), and the exact underlying mechanism is still unknown. A possible explanation is that an increased amount of effectors suppressing plant defense were delivered into host cells by the type three secretion system at a higher inoculum (Alfano and Collmer, 2004), which reduced directly or indirectly the effect of *Qpm3.1* and *hopW1-1* interaction on the restriction of bacterial growth. This finding also implies that the interaction may be effective at the early stage of infection when the bacterial load is still low. Further cloning of the allele responsible for *Qpm3.1* will help elucidate the phenomenon.

The discovery of effector-associated QTLs highlights the role of bacterial effectors in delineating natural variation in plant quantitative disease resistance. Since many agriculturally important bacterial pathogens are sequenced and effector coding genes can be efficiently predicted (da Silva et al., 2002; Guttman et al., 2002; Salanoubat et al., 2002), effector-based approaches can be thus designed to scan Arabidopsis accessions for common or rare genetic variations with large effect on pathogenesis of bacterial disease, thereby providing new insights on the fundamental bacterial effector biology. The study on *Qpm3.1* also indicated that RILs and HIFs are particularly useful for QTL identification and mendelization, which will markedly facilitate subsequent positional cloning of the responsible gene.

The effector gene *hopW1-1*, first identified from strain ES4326, is narrowly distributed in a few *P. syringae* strains (Guttman et al., 2002). This effector disrupts actin cytoskeleton and inhibits endocytosis to promote bacterial virulence when delivered into plant cells (Kang et al., 2014), but elicits resistance responses and limits bacterial growth on some Arabidopsis accessions including Ws (Lee et al., 2008). Infection of Ws plants with the strain DC3000 carrying *hopW1-1* specifically triggers an enhanced accumulation of SA at the early stage of interaction; a number of genes involved in SA pathways or encoding the HopW1-interacting proteins are required for the effector-triggered resistance in Ws; two loci, on chromosome III and V, have been identified using Col × Ws F2 population (Lee et al., 2008). Nevertheless, none of these described genes, loci locate at the identified *Qpm3.1* locus, and no gene product was annotated in the region as actin-associated linking with the proposed function of HopW1-1; therefore, additional gene(s) conditions natural variation in Arabidopsis response to HopW1-1. Interestingly, by measuring the growth of DC3000 *in planta*, Dobon et al. have identified four QTLs underpinning natural variation in exogenous SA-induced resistance across Arabidopsis accessions, among which *SAQ3*, detected in Col-0xLer RILs, locates closely to *Qpm3.1* (Dobón et al., 2011). Further investigation on the possible linkage between these two loci may shed new light on the role of SA-dependent pathways in effector-triggered immunity against plant bacterial pathogens.

## Author Contributions

JF and Y-LP designed the research; QL, W-WL, and K-DP performed the research, collected and analyzed the data; QL, JF, and Y-LP wrote the manuscript. All authors read and approved the final manuscript.

## Conflict of Interest Statement

The authors declare that the research was conducted in the absence of any commercial or financial relationships that could be construed as a potential conflict of interest.
