# Supplementary material for: Genetic Interaction between Arabidopsis Qpm3.1 Locus and Bacterial Effector Gene hopW1-1 Underlies Natural Variation in Quantitative Disease Resistance to Pseudomonas Infection
Source: Front Plant Sci. 2017 May 4;8:695. doi: 10.3389/fpls.2017.00695 (PMC5415610; doi:10.3389/fpls.2017.00695)
Supplement: Supplementary file 1 [file Data_Sheet_1.docx]

**Figure S1**


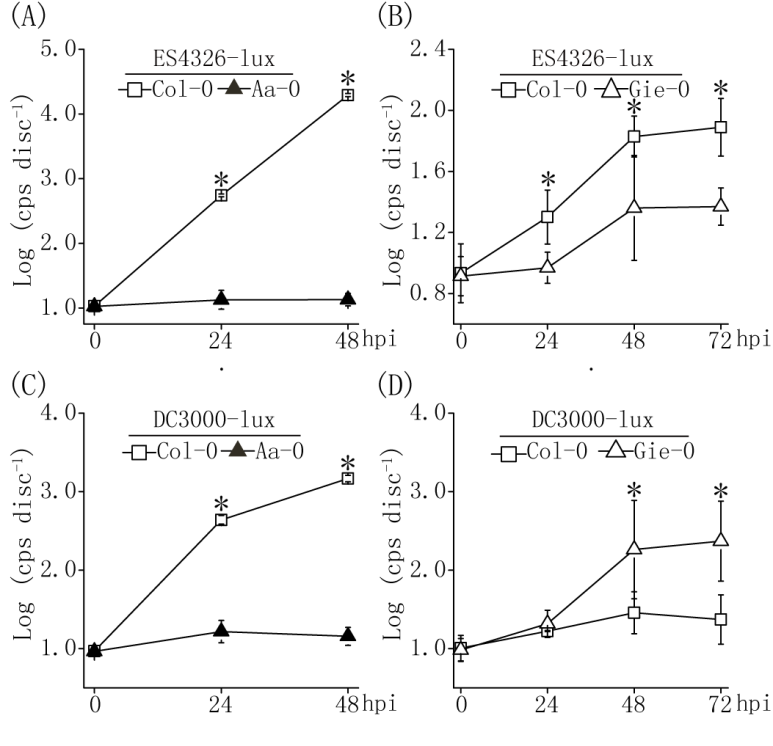


**Figure S1 Luminescence assay of growth of ES4326-lux or DC3000-lux in Col-0, Aa-0, and Gie-0 accessions.** Conditions of bacterial inoculation were the same as Fig. 1. The bacterial growth was calculated as log (cps disc^-1^). Each data shown were the average bacterial growth of at least 8 leaf discs of 5 mm in diameter excised from the inoculated leaves. The asterisks indicate significant difference between the genotypes (Student's *t* test, P＜0.05). Error bars denote standard deviations (n=8). Experiments were repeated three times with similar results. cps: photon counts per second.

**Figure S2**


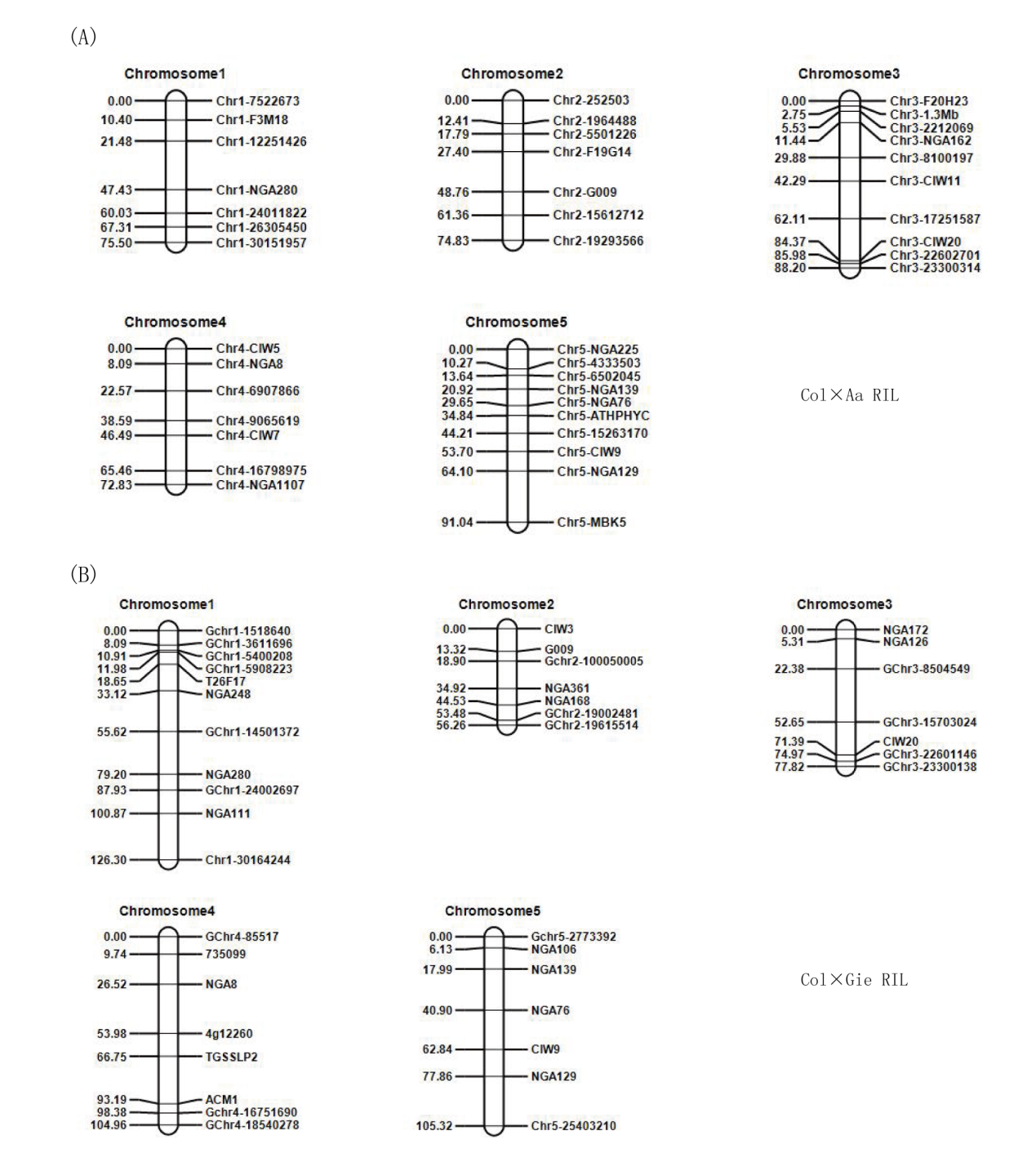


**Figure S2 Genetic maps derived from Col×Aa RILs and Col×Gie RILs.** To construct the genetic maps of Col×Aa RILs (A) and Col×Gie RILs (B), genotypic data of each RILs were analyzed by QTL Icimapping software with map function. The algorithm of nnTwoOpt was chosen to locate markers in chromosomes. Marker names and their genetic locations are shown.

**Figure S3**


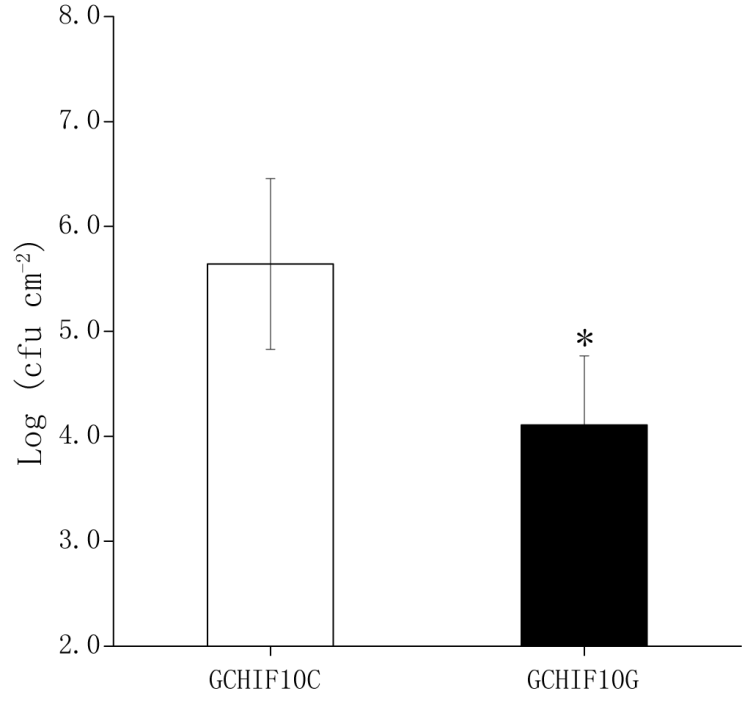


**Figure S3 Validation of the major QTL *Qpm3.1* in Gie-0 with heterogeneous inbred families (HIFs).** Forty eight individuals of a Col×Gie HIF (GCHIF10) were inoculated with ES4326-lux at OD_600_=0.0002. Genotypes of individual plants were determined with the tightly associated marker NGA76 and NGA172 and sizes of bacterial population were compared between plants with homozygous Gie-0 (GCHIF10G) and plants with Col-0 (GCHIF10C) genotypes. Levels of bacterial population were determined at 72hpi. Error bars denote standard deviations (n=8). The asterisk indicates significant difference between treatments (Student’s *t* test, P＜0.05). This experiment was repeated three times with similar results.

**Figure S4**


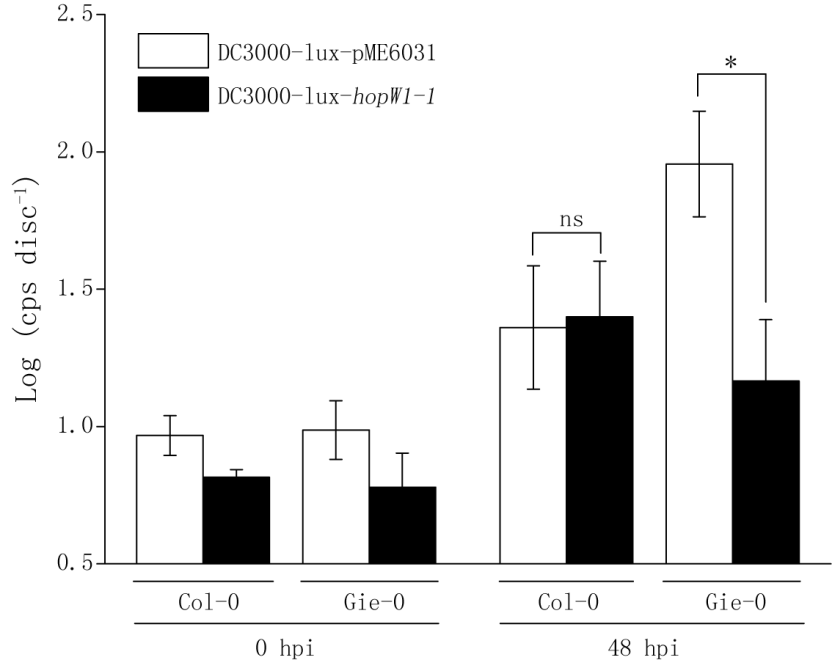


**Figure S4 The effector gene *hopW1-1* selectively reduced growth of DC3000-lux in Gie-0 but not Col-0 plants.** Plants were pressure inoculated with bacteria at OD_600_=0.0006. Bacterial growth was determined by measuring luminescence (photon counts per second, cps) of 3 leaf discs from each plant and at least 4 plants were used in each treatment. Error bars denote standard deviations (n=12). The asterisk indicates significant difference between treatments (Student’s *t* test, P＜0.05); “ns” denotes no significant difference. This experiment was repeated three times with similar results.

**Figure S5**


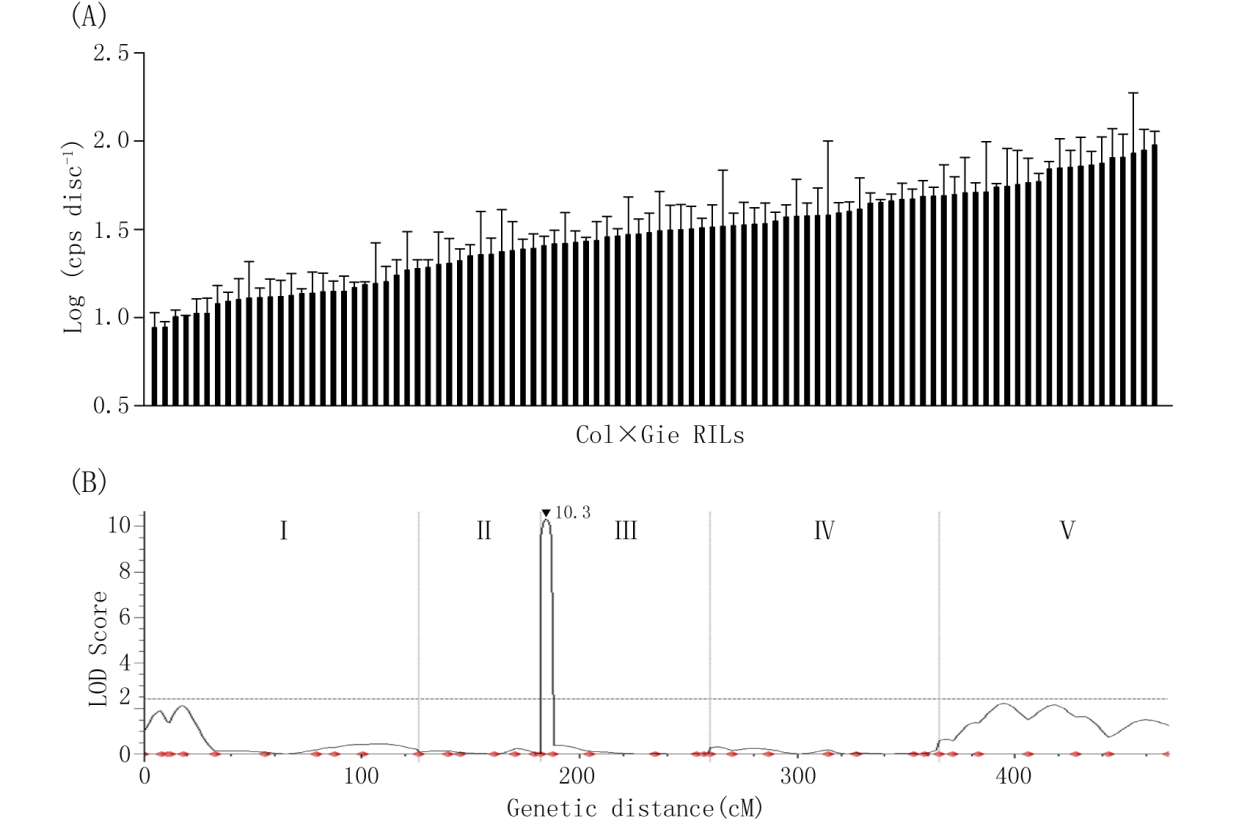


**Figure S5 The major QTL *Qpm3.1* conferred *hopW1-1* dependent resistance variance in Col×Gie RILs.** (A) Growth distribution of DC3000-lux-*hopW1-1* in Col×Gie RILs. Plant lines were pressure inoculated at OD_600_=0.0006. Leaf discs from 4 leaves per line were taken to measure luminescence (photon counts per second, cps) at 48hpi. The experiment was repeated three times and the average levels of luminescence of each line from three experiments are shown. Error bars denote standard deviations (n=3). (B) *Qpm3.1* was detected controlling resistance to DC3000-lux-*hopW1-1* in Col×Gie RILs. QTL analysis were based on genotypic data and levels of bacterial luminescence of the Col×Gie RILs. The curved line indicates likelihood statistics (scores of likelihood of odds，LOD) for the positions of QTLs and the significance threshold at 95% conﬁdence interval calculated by permutation tests (n=1000) is plotted as the horizontal line. I-V denote five individual linkage groups. Positions of the markers used for analysis are indicate as small dots. The peak of LOD scores is indicated by filled triangle along with the value.

**Figure S6**


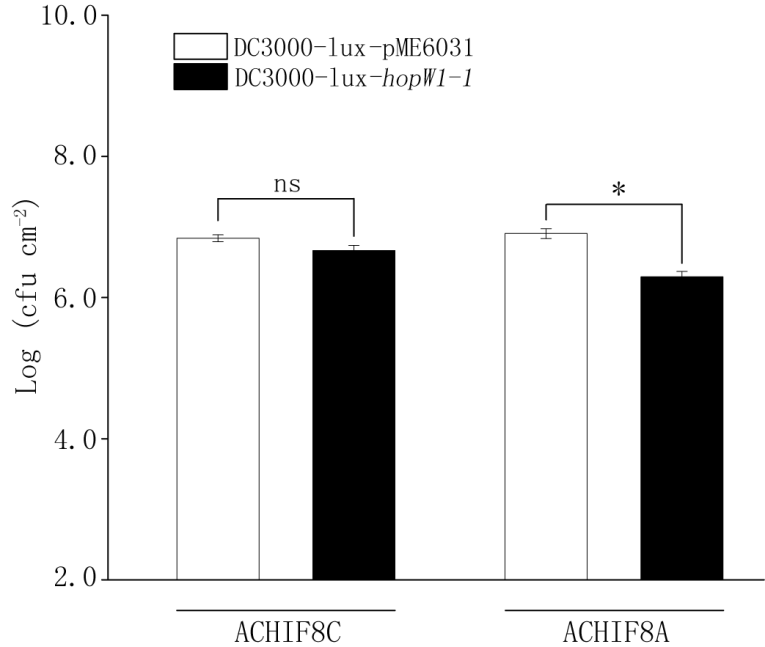


**Figure S6 The interaction between *hopW1-1* and *Qpm3.1* was influenced by bacterial inocula concentration.** Individuals of ACHIF8 were inoculated with DC3000-lux-*hopW1-1* and DC3000-lux-pME6031 at OD_600_=0.03. The inoculated plants were covered to keep high relative humidity. Genotypes of individual plants were determined with the tightly associated marker 1.3 Mb and sizes of bacterial population were compared between plants with homozygous Aa-0 (ACHIF8A) and plants with Col-0 (ACHIF8C) genotypes. Levels of bacterial population were determined at 48hpi. Data shown are means±s.d. (n=8). The asterisk indicates significant difference between treatments (Student’s *t* test, P＜0.05). “ns” denotes no significant difference. This experiment was repeated twice with similar results.
