# Supplementary material for: Genetic Interaction between Arabidopsis Qpm3.1 Locus and Bacterial Effector Gene hopW1-1 Underlies Natural Variation in Quantitative Disease Resistance to Pseudomonas Infection
Source: Front Plant Sci. 2017 May 4;8:695. doi: 10.3389/fpls.2017.00695 (PMC5415610; doi:10.3389/fpls.2017.00695)
Supplement: Supplementary file 3 [file Data_Sheet_3.docx]

**Table S2** Markers used for genotyping the Col×Aa RILs

| **Marker name** | **Forward primer (5'-3')** | **Reverse primer (5'-3')** | **Type** | **Enzyme** | **Chr.** |
| --- | --- | --- | --- | --- | --- |
| **7522673** | AGGTCGAATAAATTAAGAGGAATT | TTCTAGAAGAAAAAAATCGTAAAT | dCAPs | *EcoR*I | Ⅰ |
| **F3M18** | GTACAGGACATGTTTATGAAGC | CTTCATCGTTTCAATATTTTGCA | SSLP | — | Ⅰ |
| **12251426** | CTAATCAAGCCAAAATAAATTAAAG | CACAAATAAGGTTATATATGTCATT | dCAPs | *Hind*III | Ⅰ |
| **NGA280** | GGCTCCATAAAAAGTGCACC | CTGATCTCACGGACAATAGTGC | SSLP | — | Ⅰ |
| **24011822** | GGTTTATTCTCTTTTTTTACATTTTGA | TTGAAAGAATTAATACTGATACTGC | dCAPs | *EcoR*I | Ⅰ |
| **26305450** | TTTCGAATCATCAACCTATAAGCT | TACTCACATTTTCTCCGTCGTA | dCAPs | *Hind*III | Ⅰ |
| **30151957** | TCTCTGTCTTCCCTAATTTCTGA | TTGGTATCTCAAGTAATGGTAGA | dCAPs | *EcoR*I | Ⅰ |
| **252503** | TATCAACATAATGAAAAAAAAAGCT | ACTTACTTATCCTCAGCAGTTG | dCAPs | *Hind*III | Ⅱ |
| **1964488** | ATTACCTTGATCATTCAAACCGAAT | ATTGATGAGTCCTAGCAATGAAAG | dCAPs | *EcoR*I | Ⅱ |
| **5501226** | CCAAACTAGAAGAGGAAAAGCT | GCAGTTCATTACCATAGCCTTT | dCAPs | *Hind*III | Ⅱ |
| **F19G14** | TCCAATAGTTGGTCCACCGA | ACACCACGTTGGTTCTCTCT | SSLP | — | Ⅱ |
| **G009** | AACTTACATTCTTCAATCCTTCG | TGACTAGAGTGTATTTGATGTGG | SSLP | — | Ⅱ |
| **15612712** | TATAAACCCTAAAATCCAAAAGCT | CCATTTAAATCATATTGTTTCAGG | dCAPs | *Hind*III | Ⅱ |
| **19293566** | CTTAGCTTGACTTGAGAAATTAAAG | TAATCTTCGGGTGCTCTGTTTC | dCAPs | *Hind*III | Ⅱ |
| **F20H23** | ACTCTACTCAGCGGACTTTGTG | TGGCTTCAGCAGCATTCATGTT | SSLP | — | Ⅲ |
| **1.3Mb** | GAAGGTCCAAATTGACAACGAC | CCTTTTATCTCATTCTCCTCC | SSLP | — | Ⅲ |
| **2212069** | GCAAGCTCATGATTCCAATCTTAG | AAAATATCCCTCAATTGATGGTAT | dCAPs | *EcoR*I | Ⅲ |
| **NGA162** | CTCTGTCACTCTTTTCCTCTGG | CATGCAATTTGCATCTGAGG | SSLP | — | Ⅲ |
| **8100197** | GAAATGGACCATAAACTATTCGATA | TCTCTTCTAATTTCGCCCACTTT | dCAPs | *EcoR*V | Ⅲ |
| **CIW11** | CCCCGAGTTGAGGTATT | GAAGAAATTCCTAAAGCATTC | SSLP | — | Ⅲ |
| **17251587** | ATAGCAACACATTGAGTTAATAAAGC | TATAAAAGGAGTTTCAACGGATTC | dCAPs | *Hind*III | Ⅲ |
| **CIW20** | CATCGGCCTGAGTCAACT | CACCATAGCTTCTTCCTTTCTT | SSLP | — | Ⅲ |
| **22602701** | TCAGACACAAACTTAGCCAGGA | TATACTCGTGGTCCGGTTTATG | dCAPs | *EcoR*I | Ⅲ |
| **23300314** | AATTTGGTGAAGTATAAAAAGAATT | GGTGTGCCAATTGAGAGGAC | dCAPs | *EcoR*I | Ⅲ |
| **CIW5** | GGTTAAAAATTAGGGTTACGA | AGATTTACGTGGAAGCAAT | SSLP | — | Ⅳ |
| **NGA8** | TGGCTTTCGTTTATAAACATCC | GAGGGCAAATCTTTATTTCGG | SSLP | — | Ⅳ |
| **6907866** | ACGTCATCGGTTTCTTTAGAGATAT | TCGGACTCAAAATCTGACAAACA | dCAPs | *EcoR*V | Ⅳ |
| **9065019** | CGCCTCTATCTGTTTTTAAAAGC | GTTTCAACTTGGTATAGTATAATTT | dCAPs | *Hind*III | Ⅳ |
| **CIW7** | AATTTGGAGATTAGCTGGAAT | CCATGTTGATGATAAGCACAA | SSLP | — | Ⅳ |
| **16798975** | TATTGCAGGTGATTGATTATCTTCG | CACAATTAGACCTTCCACTGATG | dCAPs | *EcoR*V | Ⅳ |
| **NGA1107** | CGACGAATCGACAGAATTAGG | GCGAAAAAACAAAAAAATCCA | SSLP | — | Ⅳ |
| **NGA225** | TCTCCCCACTAGTTTTGTGTCC | GAAATCCAAATCCCAGAGAGG | SSLP | — | Ⅴ |
| **4333503** | AATTTGTCTAATTTGCATCCGA | GGGAAGCTACACATGAAGGTC | dCAPs | *EcoR*I | Ⅴ |
| **6502045** | TGAACTTCCATTCTCTCCAAAAAG | GCTCTCACAATCTCAGGCTTC | dCAPs | *Hind*III | Ⅴ |
| **NGA139** | GGTTTCGTTTCACTATCCAGG | AGAGCTACCAGATCCGATGG | SSLP | — | Ⅴ |
| **NGA76** | AGGCATGGGAGACATTTACG | GGAGAAAATGTCACTCTCCACC | SSLP | — | Ⅴ |
| **ATHPHYC** | CTCAGAGAATTCCCAGAAAAATCT | AAACTCGAGAGTTTTGTCTAGATC | SSLP | — | Ⅴ |
| **15263170** | ATTCTGGCTAGTCTTATGAAT | ATGCTCTTTGTGATCCCCTC | dCAPs | *EcoR*I | Ⅴ |
| **CIW9** | CAGACGTATCAAATGACAAATG | GACTACTGCTCAAACTATTCGG | SSLP | — | Ⅴ |
| **NGA129** | CACACTGAAGATGGTCTTGAGG | TCAGGAGGAACTAAAGTGAGGG | SSLP | — | Ⅴ |
| **MBK5** | GAGCATTTCACAGAGACG | ATCACTGTTGTTTACCATTA | SSLP | — | Ⅴ |

**Table S3** Markers used for genotyping the Col×Gie RILs

| **Marker name** | **Forward primer (5'-3')** | **Reverse primer (5'-3')** | **Type** | **Enzyme** | **Chr.** |
| --- | --- | --- | --- | --- | --- |
| **1518640** | CCATATACGAAGCCCAGAAACAAA | CCTCCTCTTCCTTCTTCGTACTCTC | dCAPs | *Hind*III | Ⅰ |
| **3611696** | CGGAATTCAGCTCCTGAAAGATCT | TCTGTGGAGGAATAACATTAGTAA | dCAPs | *Pst*I | Ⅰ |
| **5400208** | GCTTTCTTTTTTTCAATAGAAGCT | GAGAGTTGTGATCAAACAAACC | dCAPs | *Hind*III | Ⅰ |
| **5908223** | GAGGTATCTTCCTATCACTCAA | CGACCTATGTGTGCTATAAAGC | dCAPs | *Hind*III | Ⅰ |
| **T26F17** | ATGATGTTAATGTGATTTAGTTAG | CAGTTGCACTTGTTGTAAACATG | SSLP | — | Ⅰ |
| **NGA248** | TCTGTATCTCGGTGAATTCTCC | TACCGAACCAAAACACAAAGG | SSLP | — | Ⅰ |
| **14501372** | TGGCTTGACTTAGTGACTTCT | CATTCTGAATCAAACACAAAGCT | dCAPs | *Hind*III | Ⅰ |
| **NGA280** | GGCTCCATAAAAAGTGCACC | CTGATCTCACGGACAATAGTGC | SSLP | — | Ⅰ |
| **24004697** | ATTTTCATATTGTACTCTATTAGAA | ATCTTTTAAGAACGATCCATATGAT | dCAPs | *EcoR*I | Ⅰ |
| **NGA111** | TGTTTTTTAGGACAAATGGCG | CTCCAGTTGGAAGCTAAAGGG | SSLP | — | Ⅰ |
| **30164244** | GTATTGTTCACAAAGAAAGCT | CTTAGTTCAAAGTTGTCAAAT | dCAPs | *Hind*III | Ⅱ |
| **CIW3** | GAAACTCAATGAAATCCACTT | TGAACTTGTTGTGAGCTTTGA | SSLP | — | Ⅱ |
| **10050005** | ACCCTAATATCTTCGCCTGC | GGTCGACTTTACCTGTGTCT | dCAPs | *EcoR*I | Ⅱ |
| **G009** | AACTTACATTCTTCAATCCTTCG | TGACTAGAGTGTATTTGATGTGG | SSLP | — | Ⅱ |
| **NGA361** | ACATATCAATATATTAAAGTAGC | AAAGAGATGAGAATTTGGAC | SSLP | — | Ⅱ |
| **NGA168** | GAGGACATGTATAGGAGCCTCG | TCGTCTACTGCACTGCCG | SSLP | — | Ⅱ |
| **19002481** | AAAACAAGGAAAGACAGTTTGTAAG | AACGAGAGAGAACTGAAACGGG | dCAPs | *Hind*III | Ⅱ |
| **19615514** | ACATGGCCAGGATGCTCGAATT | CACGTGTGTATCAAATACCCAAAA | dCAPs | *EcoR*I | Ⅱ |
| **NGA172** | CATCCGAATGCCATTGTTC | AGCTGCTTCCTTATAGCGTCC | SSLP | — | Ⅲ |
| **NGA126** | CAAGAGCAATATCAAGAGCAGC | GAAAAAACGCTACTTTCGTGG | SSLP | — | Ⅲ |
| **8504549** | AAAACCACTCACACTACCACAAT | CTCAGTATAGAAATTCATACGAAGCT | dCAPs | *Hind*III | Ⅲ |
| **15703024** | CCTATCACCTTAATTATGTTGAATT | CTGTCGTACTCCAAAGCGTGTG | dCAPs | *EcoR*I | Ⅲ |
| **CIW20** | CATCGGCCTGAGTCAACT | CACCATAGCTTCTTCCTTTCTT | SSLP | — | Ⅲ |
| **22601146** | CGTAATATAACATTCTGGAAAGCT | CAAGATTTGTGATCATGACTTCGT | dCAPs | *Hind*III | Ⅲ |
| **23300138** | TTACAGTTGTTGGTGAGGTAAGC | ATCGAGCGGTACAGGTGAGACA | dCAPs | *Hind*III | Ⅲ |
| **85517** | ATGTCCCTCTATTTTATTATAAAGC | AGAAAAGGCAAGTGGGTTTGGATG | dCAPs | *Hind*III | Ⅳ |
| **735099** | TTTTACGATTTTAATTTATTAAGCT | GATCCTAATTAGAATGATAAAGA | dCAPs | *Hind*III | Ⅳ |
| **NGA8** | TGGCTTTCGTTTATAAACATCC | GAGGGCAAATCTTTATTTCGG | SSLP | — | Ⅳ |
| **4G12260** | CTGGCTGGGTCAAGGAAACT | TGAGAAGGCACAAAAGAGGAGA | SSLP | — | Ⅳ |
| **TGSSLP2** | GGGAGATTAAAGAAGCCTTTGC | GTGCGGTTAACTGTTCGGTTACC | SSLP | — | Ⅳ |
| **ACM1** | GTGTTTGTATGCGTCGACAAAGAAG | AAGCAAGATTGGTTCTGCGTAAACC | SSLP | — | Ⅳ |
| **16751690** | CATCAAAATCTTGGACCTCTGCA | TTAAACTCTTGAGTACTTTTTCGAA | dCAPs | *Pst*I | Ⅳ |
| **18540278** | CTCCTCAAATGCATCAGATCTAAG | TTCTGCTTCAGATGAGTATTGTTCT | dCAPs | *Hind*III | Ⅳ |
| **2773392** | GGATCCCTAACTGTAAAATCCC | TACCGTCAATTTCATCGCC | dCAPs | *Hind*III | Ⅴ |
| **NGA106** | TGCCCCATTTTGTTCTTCTC | GTTATGGAGTTTCTAGGGCACG | SSLP | — | Ⅴ |
| **NGA139** | GGTTTCGTTTCACTATCCAGG | AGAGCTACCAGATCCGATGG | SSLP | — | Ⅴ |
| **NGA76** | AGGCATGGGAGACATTTACG | GGAGAAAATGTCACTCTCCACC | SSLP | — | Ⅴ |
| **CIW9** | CAGACGTATCAAATGACAAATG | GACTACTGCTCAAACTATTCGG | SSLP | — | Ⅴ |
| **NGA129** | CACACTGAAGATGGTCTTGAGG | TCAGGAGGAACTAAAGTGAGGG | SSLP | — | Ⅴ |
| **25403210** | CCACTCCACAAGTTGACAAGCT | TCAGGAGGAACTAAAGTGAGGG | dCAPs | *Hind*III | Ⅴ |
